# All-Trans Retinoic Acid Ameliorates Myocardial Ischemia/Reperfusion Injury by Reducing Cardiomyocyte Apoptosis

**DOI:** 10.1371/journal.pone.0133414

**Published:** 2015-07-17

**Authors:** Zhengbin Zhu, Jinzhou Zhu, Xiaoran Zhao, Ke Yang, Lin Lu, Fengru Zhang, Weifeng Shen, Ruiyan Zhang

**Affiliations:** 1 Department of Cardiology, Rui Jin Hospital, School of Medicine, Shanghai Jiao Tong University, Shanghai, 200025, PR China; 2 Institute of Cardiovascular Diseases, School of Medicine, Shanghai Jiao Tong University, Shanghai, 200025, PR China; Virginia Commonwealth University, UNITED STATES

## Abstract

Myocardial ischemia/reperfusion (I/R) injury interferes with the restoration of blood flow to ischemic myocardium. Oxidative stress-elicited apoptosis has been reported to contribute to I/R injury. All-trans retinoic acid (ATRA) has anti-apoptotic activity as previously reported. Here, we investigated the effects and the mechanism of action of ATRA on myocardial I/R injury both *in vivo* and *in vitro*. *In vivo*, ATRA reduced the size of the infarcted area (17.81±1.05% *vs*. 24.41±1.03%, *P*<0.05) and rescued cardiac function loss (ejection fraction 46.42±6.76% *vs*. 37.18±4.63%, *P*<0.05) after I/R injury. Flow-cytometric analysis and TUNEL assay demonstrated that the protective role of ATRA on myocardial I/R injury was related to its anti-apoptotic effects. The anti-apoptotic effects of ATRA were associated with partial inhibition of reactive oxygen species (ROS) production and significantly less phosphorylation of mitogen-activated protein kinases (MAPKs) including p38, JNK, and ERK. Western blot analysis also revealed that ATRA pre-treatment increased a disintegrin and metalloproteinase domain-containing protein 10 (ADAM10) expression (0.65 ± 0.20 *vs*. 0.41±0.02 *in vivo*) and reduced the level of receptor for advanced glycation end-products (RAGE) (0.38 ± 0.17 *vs*. 0.52 ± 0.11 *in vivo*). Concomitantly, the protective role of ATRA on I/R injury was not observed in RAGE-KO mice. The current results indicated that ATRA could prevent myocardial injury and reduced cardiomyocyte apoptosis after I/R effectively. One possible mechanism underlying these effects is that ATRA could increase ADAM10 expression and thus cleave RAGE, which is the main receptor up-stream of MAPKs in myocardial I/R injury, resulting in the down-regulation of MAPK signaling and protective role on myocardial I/R injury.

## Introduction

Ischemic heart disease is one of the leading causes of death worldwide. The current standard therapy for acute myocardial infarction is myocardial reperfusion [1]. However, this process can itself cause injury, known as myocardial ischemia/reperfusion (I/R) injury. It is unclear whether myocardial reperfusion does more harm than good [2]. In animal models of myocardial I/R, reperfusion itself can cause up to 30–40% of the total damage [3].

The precise pathophysiology of myocardial I/R injury remains to be established. Several mechanisms have been proposed: 1) Some cardiomyocytes cannot be rescued despite reperfusion [4]; 2) the so-called no-reflow phenomenon, which refers to decreased regional myocardial blood flow after reperfusion [5]; and 3) most importantly, generation of reactive oxygen species (ROS) after reperfusion, which can induce inflammation and trigger myocardial cell apoptosis [6,7].

All-trans retinoic acid (ATRA) is derived from vitamin A and it possesses various activities. ATRA has been markedly successful with acute promyelocytic leukemia (APL) [8]. ATRA binds to retinoid X receptors (RXRs) and nuclear retinoic acid receptors (RARs). This can either induce or repress transcription of nearby genes [9], and thereafter, modulate a large number of essential biological processes including cell differentiation [10], proliferation [11], and apoptosis [12]. ATRA has been demonstrated to have anti-apoptotic effects on human neuronal cells, and protective effects against liver I/R injury [13]. However, current literature does not indicate whether ATRA can prevent myocardial I/R injury. The purpose of this study was to determine the effects of ATRA on myocardial I/R injury, and to identify the mechanisms by which ATRA may ameliorate myocardial I/R injury.

## Materials and Methods

### Animals

Pathogen-free, 12-week-old male C57BL/6 mice (SLACCAS, Shanghai, China, n = 12) and receptor for advanced glycation end-products (RAGE) knock-out (KO) mice (provided by Prof. Ann Marie Schmidt’s lab, New York University, New York, NY, U.S., n = 12) were kept in air-filtered units at 21 ± 2°C and 50 ± 15% relative humidity during the entire experiment. Mice were provided with rodent food and sterile water ad libitum. Standards from the Guide for the Care and Use of Laboratory Animals (NIH Publication no. 85–23, revised 1996) were followed. The study protocol was approved by the Animal Use and Care Committee (Shanghai Jiao Tong University School of Medicine, document number: *SYKX-2008-0050)*.

### Surgical procedure and experimental design

C57BL/6 mice were randomly divided into sham operated (Sham, n = 3), I/R injury (I/R, n = 6), and I/R injury with ATRA treatment group (ATRA+I/R, n = 6). RAGE-knock out (RAGE-KO) mice were randomly divided into sham operated (RKO, n = 3), I/R injury (RKO-I/R, n = 6), and I/R injury with ATRA treatment group (ATRA+RKO-I/R, n = 6). Experimental I/R injury was induced by 30 min of transient myocardial ischemia effected by occlusion of the left anterior descending artery (LAD), followed by reperfusion. Briefly, mice were intraperitoneally injected with 5 mg/kg xylazine and 100 mg/kg ketamine, and then subjected to tracheal intubation ventilated with room air using a rodent mini-ventilator, placed in the right lateral decubitus position (Harvard Apparatus, Holliston, MA, U.S.). Each mouse was subjected to left thoracotomy, and their hearts were accessed between the third and fourth intercostal space. An 8–0 Prolene suture was placed around the LAD immediately after the bifurcation of the major left coronary artery and ligated for 30 min monitored by electrocardiography. The ligature was then removed, and blood flow was restored. The chest cavity was then closed. Mice were allowed to recover on a constant temperature plate (37°C). Mice were monitored every 2 hours till 12 hours after surgery, every 6 hours till 2 days after surgery, and once per day for the remainder of the experiment. To evaluate the effect of ATRA, groups of mice were treated with ATRA (10 mg/kg in 0.05 mL peanut oil) administered *via* i.p. 30 min before surgery (day 0), and once per day for 7 days after reperfusion (day 1-day 7).

### Echocardiography

Mice were subjected to M-mode echocardiography using a Vevo 770 small-animal echocardiography analysis system at baseline and at day 8 post I/R prior to mouse death (Visual Sonics Inc., Toronto, Canada). Briefly, the mice were anesthetized using 1–1.5% isoflurane, administered by inhalation. The upper sterna and subxiphoid areas were shaved and lubricated to improve acoustic coupling. Each electrode was attached to a limb. Using a 30 MHz transducer, the cardiac cycle parameters were recorded by performing M-mode echocardiography of the left ventricle in the parasternal long-axis view. The stable beat rates of all animals indicated the level of anesthesia state during the operation (300–350 bpm). The function of the left ventricle was calculated by calculation of cardiac output, diastolic volume, systolic volume, and ejection fraction (EF). All measurements were manually obtained by the same observer. After measurements, mice were allowed to recover on a constant temperature plate (37°C).

### Pathologic examination

Infarcts were measured using triphenyl tetrazolium chloride (TTC) staining. Briefly, mice were sacrificed by sodium pentobarbital overdose (200 mg/kg, intraperitoneally) at day 8 post I/R after echocardiography analysis, and their hearts were extracted promptly and mounted using a Langendorff apparatus (130105EZ Radnoti, Monrovia, CA, U.S.). The coronary arteries were perfused with PBS. Blood was washed out, and the left anterior coronary artery was re-occluded. An approximately 1mL bolus of 2% Evans blue dye was injected into the heart at the aorta until the heart turned blue. The heart was then removed and cut into 5 transverse slices. These were then incubated at 37°C for 20 min in 1% TTC solution. Slices were photographed, risk and the infarcted section of the myocardium stained by TTC or Evans blue was measured using computer morphometry and ImagePro Plus 6.0 software (Media Cybernetics, Silver Spring, MD, U.S.). Representative images including papillary muscle of left ventricle were presented from different groups.

### Cell culture

H9c2, a cell line derived from rat cardiomyocytes, was obtained from the China Science Academy Cell Bank (Shanghai, China). Cells were cultured in high-glucose Dulbecco’s Modified Eagle Medium (DMEM) with 100 μg/ml streptomycin, 100 μg/ml penicillin (Gibco, NY, U.S.), and 10% fetal bovine serum (FBS) and in a humidified incubator at 37°C with 5% CO_2_.

### *In vitro* I/R injury model

A simulated I/R cell model (hypoxia/reoxygenation, H/R) was performed, with slight modifications from previous descriptions [14,15]. Briefly, H9c2 cells were seeded into culture plates and incubated for 24 h for cells to attach. Then the culture medium was replaced. Cells were treated with different concentrations of ATRA (Sigma, MO, U.S.) for 24 h. Cells were incubated in a 37°C modular incubator chamber (Billups-Rothenberg, CA, U.S.) with 95% oxygen and 5% CO_2_. Nitrogen gas was flushed into the incubator to decrease oxygen to 1%. Cells were subjected to 6 h of hypoxia followed by re-oxygenation by incubation in DMEM culture medium with 1% FBS at 37°C with 95% oxygen and 5% CO_2_ for an additional hour. Cells were then used for later experiments.

### Cell proliferation assay

A previous study revealed that ATRA could facilitate the proliferation of H9c2 cells in normal condition [16]. In the present study, the effect of ATRA on H9c2 cell viability after H/R injury was evaluated using a WST-1 assay. Briefly, 1 × 10^4^ cells were seeded, per well, in 96-well culture plates (passage 6~10, ~3 × 10^4^ cells per cm^2^) and incubated for 24 h. ATRA, in 10-fold serial dilutions from 100 μM to 1 nM in DMSO, was added to the culture medium and allowed to incubate for 24 h. DMSO equivalent to ATRA at the highest concentration served as an H/R group. Control refers to cells with neither H/R injury nor ATRA treatment. After H/R treatment, 10 μM of WST-1 cell proliferation assay reagent was added to each well and allowed to incubate for 3.5 h (Roche Applied Science, Mannheim, Germany). Absorbance was read at 450 nm using an enzyme-linked immunosorbent assay (ELISA) microplate reader (BioTek, VT, U.S.).

### TUNEL assay

The effect of ATRA on I/R-induced myocardial apoptosis was assessed using terminal deoxynucleotidyl transferase dUTP nick end labeling (TUNEL, Roche, Basel, Switzerland) assay both *in vivo* and *in vitro*. *In vivo*, heart tissues with I/R injury from I/R and ATRA+I/R group were fixed in 4% paraformaldehyde at room temperature for 1 day. Tissues were dehydrated through a graded ethanol series, embedded in paraffin blocks, cut into ultrathin sections (5 μm), and deparaffinized. *In vitro*, H9c2 cells were treated with ATRA for 24 h and followed by H/R treatment. Cells were then fixed with 4% paraformaldehyde for 10 min. Both H9c2 cells and heart tissues were rehydrated and then incubated with a reaction mixture containing terminal deoxynucleotidyl transferase and fluorescein isothiocyanate-conjugated dUTP in a humidified chamber for 1 h at 37°C in the dark. DNA fragmentation was detected using a fluorescence microscope and localized green fluorescence inside the nucleus of apoptotic cells. TUNEL quantification was made by counting TUNEL-positive cells using five random fields at x 200 magnification.

### Flow-cytometric analysis of apoptosis

The effect of ATRA on cell apoptosis was also determined using an Annexin V-Alexa Fluor 488 kit (Invitrogen, OR, U.S.). H9c2 cells cultured on 6-well culture plates were pre-treated and exposed to different concentrations of ATRA or a DMSO equivalent to ATRA at the highest concentration for 24 h which served as a negative control, or 1 mM H_2_O_2_ which served as an apoptosis-positive control, for 2 h. After H/R, the H9c2 cells were collected and then washed two times in ice-cold PBS. After cells were centrifuged at 300 x *g* for 5 min, the pellets were resuspended in Alexa Fluor 488-annexin V and propidium iodide binding buffer, mixed gently, and then incubated for 10 min on ice in the dark. Then samples were analyzed at 494 nm excitation wavelength using fluorescence-activated cell sorting (FACS) and a FACSAria I Cell Sorter (Becton-Dickinson, CA, U.S.). Alexa Fluor 488 emission was detected using a 520 nm band pass filter, and a > 600 nm filter was used to detect propidium iodide. The relative number of Annexin-V-positive cells in the H9c2 cells populations was measured using FACSDiva software (Becton-Dickinson, CA, U.S.). The experiment was performed in triplicate.

### Measurement of ROS production

Intracellular oxidant formation in the presence or absence of ATRA was measured in H9c2 cells loaded with 5-(and-6)-carboxy-2′,7′-dichlorodihydrofluorescein diacetate (carboxy-H2DCFDA) (Invitrogen, CA, USA), a non-fluorescent and cell-permeable analog that is converted into carboxy-2′,7′-dichlorodihydrofluorescein after intracellular deacetylation and is subsequently oxidized to highly fluorescent carboxy-dichlorofluorescein (carboxy-DCF). Cells were incubated for 15 min with 10 μM carboxy-H2DCFDA in warm Hank's Buffered Salt Solution (HBSS), then washed and immediately viewed by fluorescence microscopy.

### Western blot analysis

Proteins from cardiac samples and H9c2 cells were extracted using a ProteoJET Mammalian Cell Lysis Reagent (Fermentas, MD, U.S.) containing protease and phosphatase inhibitors. The lysate solution was centrifuged for 15 min at 4,000 x *g* at 4°C. The supernatant was transferred into new tubes. After quantification of protein was performed using BCA Protein Assay, 40 μg of total protein was electrophoresed using 10% SDS-PAGE, and transferred to PVDF membranes (Millipore, MA, U.S.). Membranes were blocked for 1 h at 4°C with 5% non-fat milk. Then they were incubated overnight at 4°C with the primary antibody (rabbit monoclonal anti-phospho-specific p38, anti-phospho-specific JNK, anti-phospho-specific ERK, anti-p38, anti-JNK, anti-ERK, anti-bcl-2, anti-cleaved caspase 3, anti-GAPDH, Cell Signaling, CA, U.S.; rabbit monoclonal anti-RAGE, anti-ADAM10, Abcam, MA, U.S.) diluted 1:1000 in TBST with 5% BSA. Membranes were washed with TBST, followed by an incubation of 1 h at room temperature with either horseradish-peroxidase-conjugated goat anti-rabbit secondary antibodies or goat anti-mouse (1:1000). The positive protein bands were developed using a chemiluminescent system and semi-quantified using Quantity One 1-D Analysis Software (Bio-Rad, CA, U.S.). GAPDH served as an internal control for normalization of protein quantity.

### Immunohistochemistry

For immunohistochemical analysis of a disintegrin and metalloproteinase domain-containing protein 10 (ADAM10), RAGE and mitogen-activated protein kinases (MAPKs) activities, heart tissues were fixed in 10% formalin at room temperature for 1 day. Samples were dehydrated through a graded ethanol series and embedded in paraffin blocks. Ultrathin sections of 5 μm were deparaffinized, rehydrated, and incubated for 5 min in 3% H_2_O_2_ to inhibit endogenous peroxidase activity. Masking of non-specific binding sites was performed by incubation in 5% BSA for 30 min. Subsequently, sections were incubated with rabbit monoclonal anti-phospho-specific p38, anti-phospho-specific JNK, anti-phospho-specific ERK, anti-RAGE, and anti-ADAM10 diluted to a ratio of 1:1,000 overnight at 4°C, and then incubated with biotin-conjugated rat anti-rabbit secondary antibody for 30 min at 37°C, and finally incubated in streptavidin-biotin complex (SABC) for 30 min at 37°C. Peroxidase activity was measured after incubation with diaminobenzidine tetrahydrochloride (DAB) as a chromagen. After washing, sections were counterstained with hematoxiylin. Observations and photographic records were made using a phase contrast microscope.

### Statistical analysis

Continuous variables were presented as mean ± standard deviation (S.D.). Categorical variables are described by counts and percentages. Wilcoxon test, Student's *t* test and *chi*-square test were used to assess statistically significant differences among different groups. A value of *P*<0.05 was considered as statistically significant. SPSS 13.0 software was used for data analysis (SPSS Inc., IL, U.S.).

## Results

### ATRA protects mice from I/R-induced loss of cardiac function

Echocardiographic analyses were shown in Table 1. All images were recorded at heart rates of 300–350 bpm and no significant differences were detected at baseline for Sham, I/R, and ATRA+I/R group. At day 8 post-I/R, I/R injury induced significant cardiac function loss (EF = 37.18±4.63% in I/R *vs*. 60.13±6.12% in Sham). Compared to the non-treated group, ATRA treatment improved cardiac input (14.93±3.54 mL/min in ATRA+I/R *vs* 12.07±3.05 mL/min in I/R, *P*<0.05), decreased LV diastolic volume (58.22±8.34 μL in ATRA+I/R *vs* 59.52±8.12 μL in I/R, *P*<0.05) and systolic volume (25.34±4.109 μL in ATRA+I/R *vs* 30.76±5.54 μL in I/R, *P*<0.05). As a result, mice treated with ATRA demonstrated a significant improvement in percent EF when compared to non-treated group (46.42±6.76% in ATRA+I/R *vs* 37.18±4.63% in I/R, *P*<0.05).

**Table 1:** Characteristics of cardiac function in Sham, I/R, and ATRA+I/R groups before and after I/R injury.

	Baseline (Day 0)			Day 8 Post- I/R injury		
	Sham group (n = 3)	I/R group (n = 6)	ATRA+I/R group (n = 6)	Sham group (n = 3)	I/R group (n = 6)	ATRA+I/R group (n = 6)
Cardiac output (mL/min)	14.91±1.93	14.51±2.82	14.79±3.18	15.23±1.05	12.07±3.05^a^	14.93±3.54^a^ ^,^ ^b^
Volume; diastolic (μL)	78.93±11.24	77.97±15.15	78.35±16.46	80.03±12.35	59.52±8.12^a^	58.22±8.34^a^
Volume; systolic (μL)	33.57±5.05	34.48±8.12	31.45±9.43	34.59±5.15	30.76±5.54^a^	25.34±4.19^a^ ^,^ ^b^
Ejection fraction (%)	60.09±3.98	60.45±6.46	59.07±4.23	60.13±3.12	37.18±4.63^a^	46.42±6.76^a^ ^,^ ^b^
Heart rate (BPM)	306±24	327±29	316±14	326±27	310±35	319±23

All data were given mean ± SD.

^a^ Relative to Sham group,

^b^ Relative to I/R group (*P*<0.05).

### ATRA treatment reduced cardiac infarct size after I/R injury

**Fig 1:**
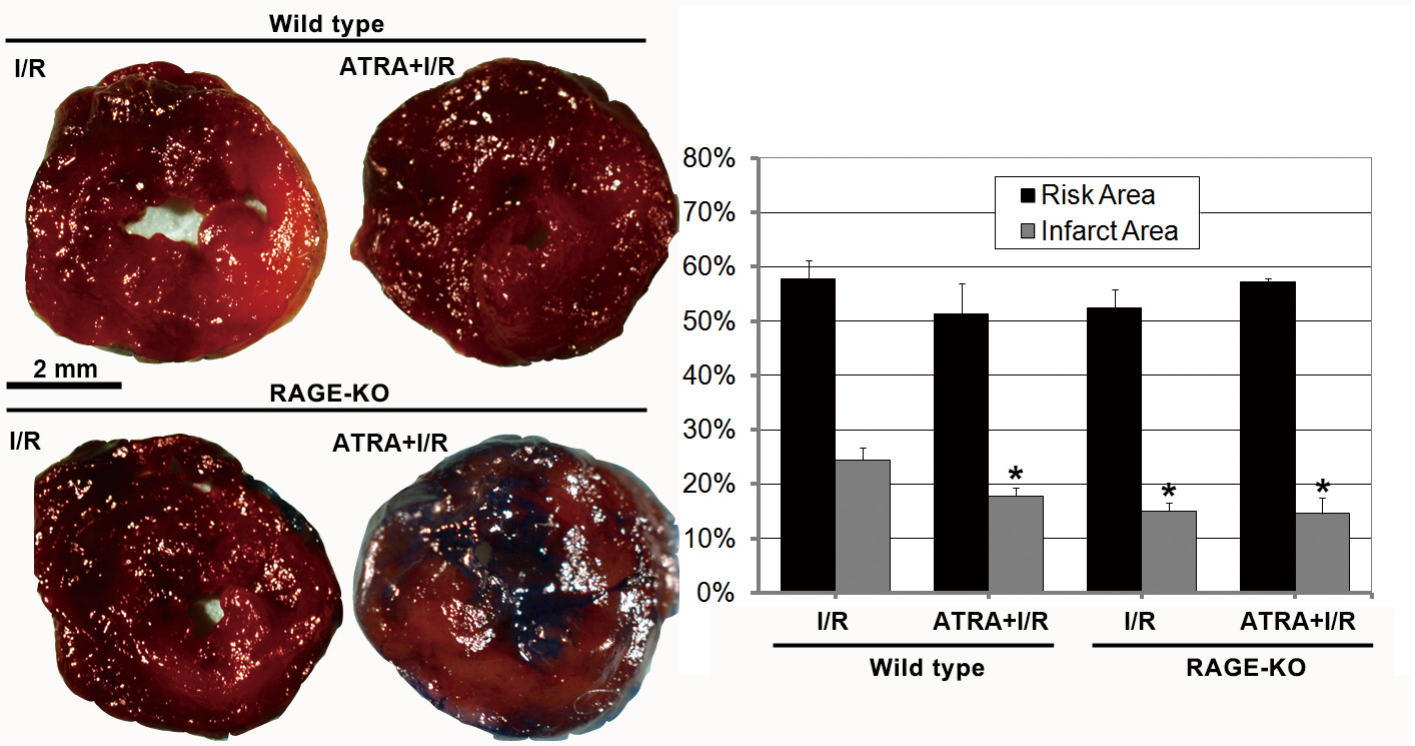
ATRA protects mice from I/R-induced cardiac injury. Images show transverse, TTC-stained sections of mouse hearts from I/R, ATRA+I/R, RKO-I/R, and ATRA+RKO-I/R groups. After I/R injury, the infarct areas were significantly smaller in ATRA+I/R group than in I/R group (n = 3 in each group). The protective role of ATRA on myocardial I/R injury was not observed in RAGE-KO mice, as the infarct areas did not differ between RKO-I/R and ATRA+RKO-I/R group (n = 3 in each group). Data is expressed as mean ± SD (^#^ *P*<0.05 *vs*. I/R group).

Cardiac infarction due to I/R was evaluated using TTC staining. Representative sections (Fig 1) demonstrated that I/R induced infarction was attenuated in ATRA-treatment group. The infarction was reduced to 17.81±1.05% in the ATRA+I/R group compared with 24.41±1.03% in I/R group (*P*<0.05).

### ATRA treatment increased cell survival after I/R injury

**Fig 2:**
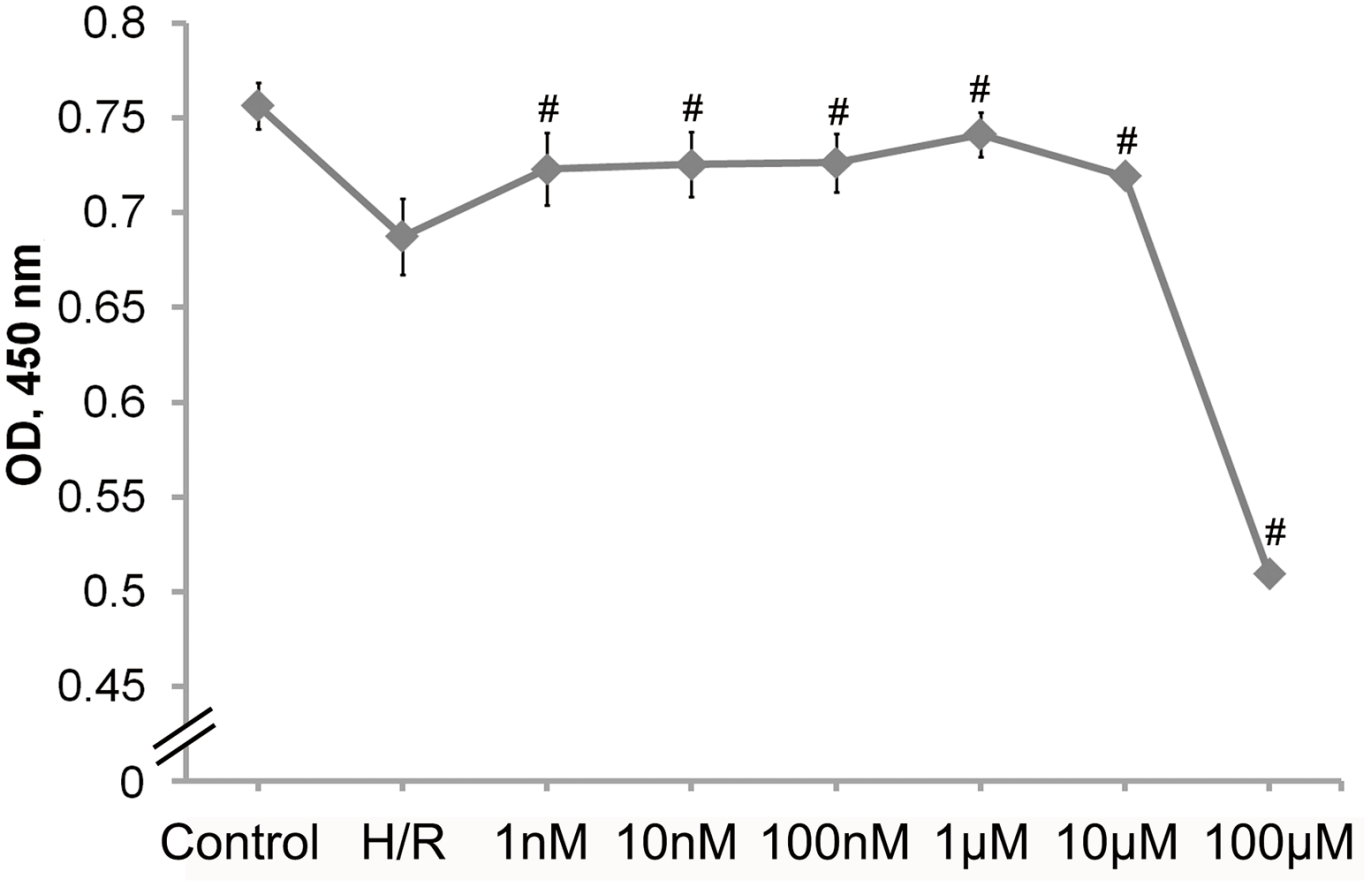
ATRA protects H9c2 cells from H/R-induced cell death. H9c2 cells subjected to H/R injury were pre-treated with various concentrations of ATRA for 24 h. Cells without H/R injury served as control. The WST-1 method was used to assess cell viability. (n = 5 in each group, ^#^ *P*<0.05 *vs*. H/R group). Data is expressed as means ± SD.

Cell survival after H/R injury was assessed using the WST-1 assay. Cells that had been subjected to a 6 h of simulated ischemia, then followed by 1 h of reperfusion, exhibited approximately 10% less cell viability (H/R *vs*. control) With ATRA treatment (1 nM to 10 μM, n = 5 in each group), H9c2 cells were significantly protected from H/R injury in a concentration-dependent manner. However, the 100 μM ATRA treatment group exhibited significant cell toxicity (Fig 2).

### ATRA protected against I/R-induced apoptosis

**Fig 3:**
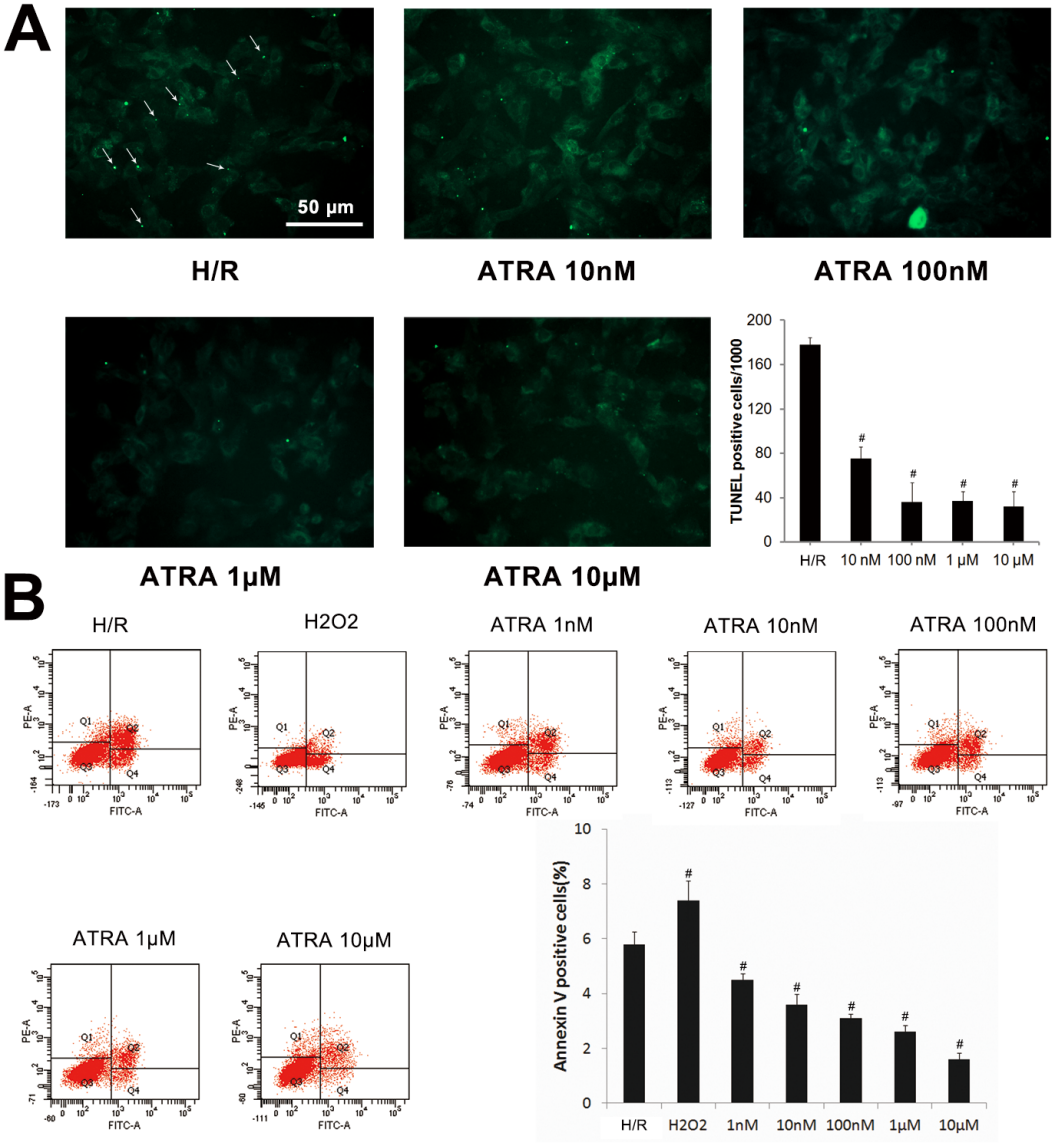
ATRA protects H9c2 cells from H/R-induced cell apoptosis. (**A**) TUNEL detection of H9c2 cells from H/R (n = 3) and ATRA (n = 3) treatment groups (×200) indicated large numbers of TUNEL-positive cells were visible in the H/R group (arrow), but were lower in the ATRA treatment group. Quantitative analysis of apoptosis revealed significantly fewer apoptotic cells in ATRA treatment groups. (**B**) After 2 h of treatment with different concentrations of ATRA for 24 h or 1 mM H_2_O_2_, H9c2 cells were subjected to H/R injury, and the relative number of annexin-V-positive cells was determined using flow cytometry (n = 3 in every group, ^#^ *P*<0.05 *vs*. H/R). Data is expressed as means ± SD.

**Fig 4:**
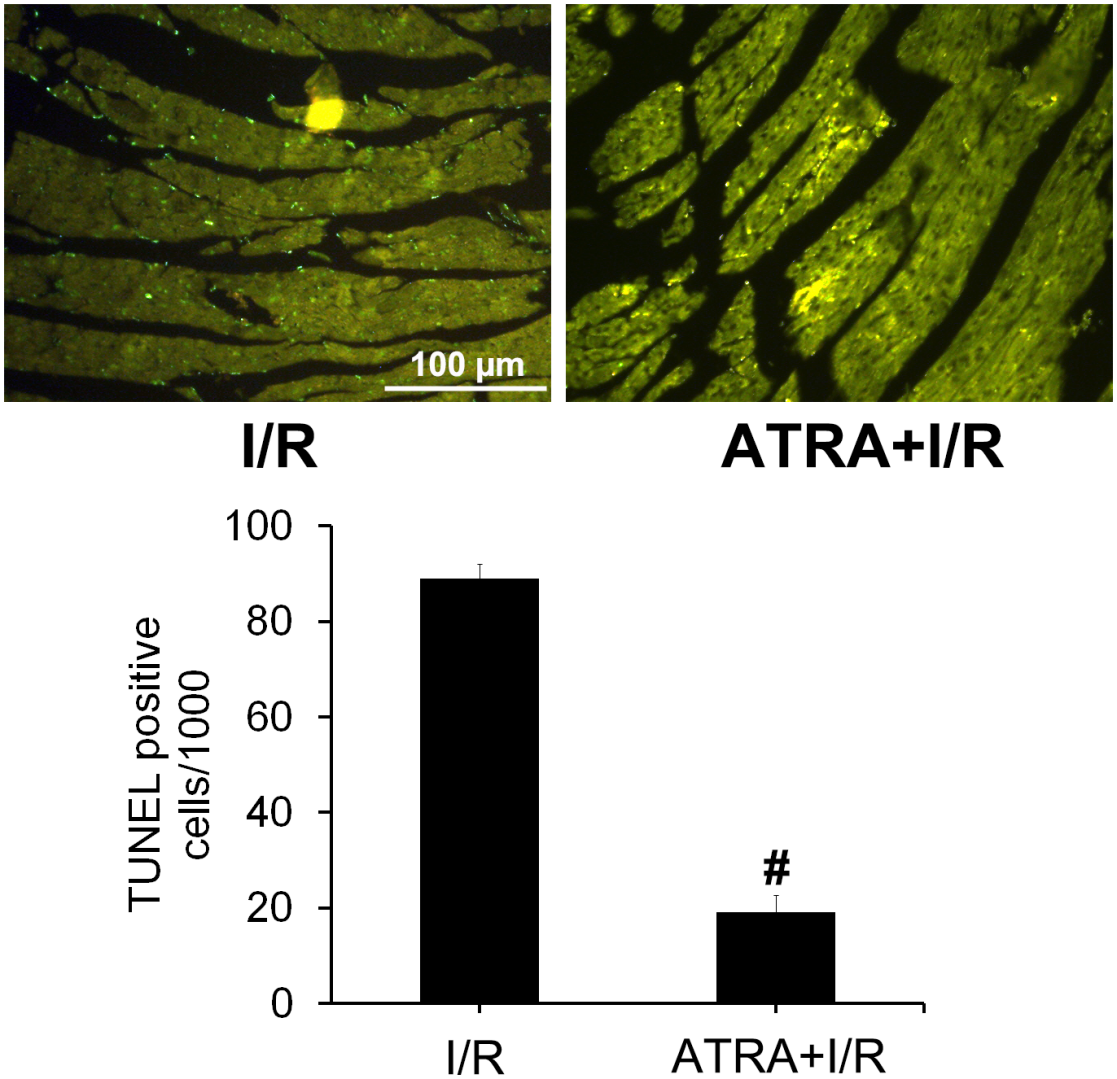
ATRA protects cardiomyocytes from I/R-induced apoptosis. (**A**) TUNEL detection of heart tissues with I/R injury from I/R and ATRA+I/R group (x200), showing that TUNEL-positive cells were prominent in I/R group, but reduced in the ATRA+I/R group. (**B**) Quantitative analysis of apoptotic cell death showed that there was significantly decreased apoptotic cells in the ATRA+I/R group compared to the I/R group (n = 3 in each group, ^#^ *P*<0.05).

**Fig 5:**
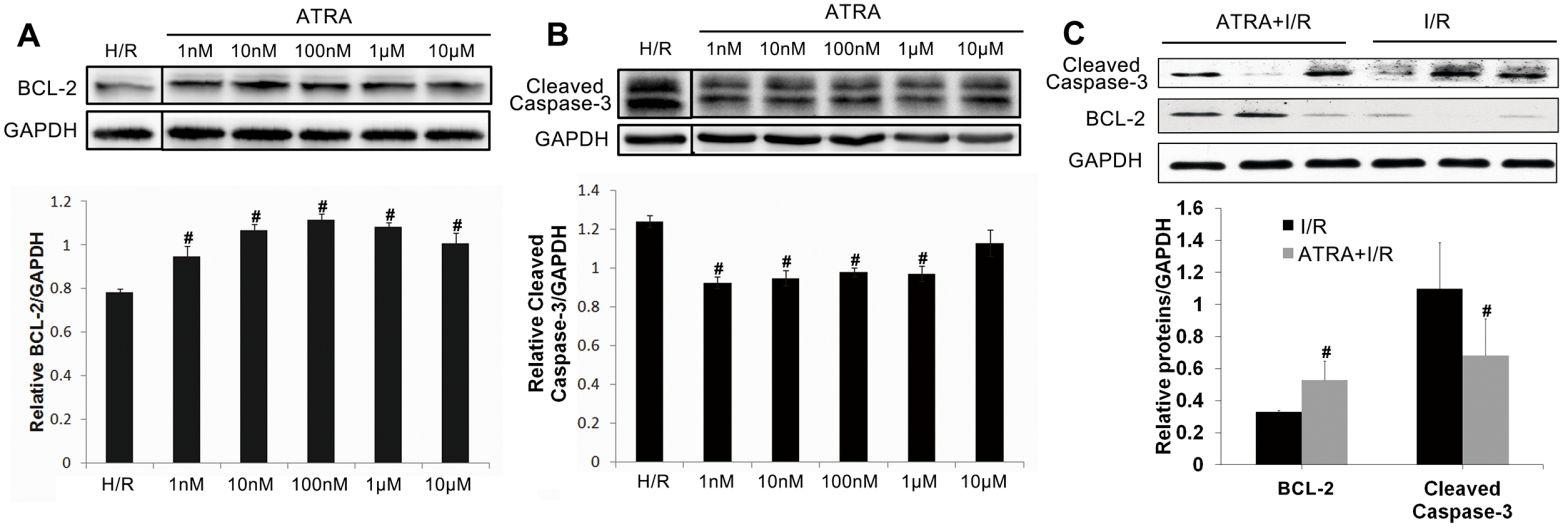
ATRA decreases cleaved caspase-3 and increases bcl-2 activities after I/R injury. (**A+B**) H9c2 cells were subjected to 24 h of pre-treatment with different concentrations of ATRA and then subjected to H/R injury. A DMSO equivalent to ATRA at the highest concentration served as a control (H/R group). Western blotting of cell lysates indicated that there was less cleaved caspase-3 protein and more bcl-2 protein in cells treated with ATRA than in controls (n = 3 in every group, ^#^ *P*<0.05 *vs*. H/R). (**C**) The *in vitro* findings were confirmed in mice cardiomyocyte after I/R injury (n = 3 in every group, ^#^ *P*<0.05 *vs*. I/R). Data is expressed as means ± SD.

To determine whether ATRA protects myocardiocytes from I/R-induced apoptosis, DNA fragmentation was compared in H9c2 cells and heart tissues with and without ATRA treatment using the TUNEL assay. As shown in Fig 3A, after H/R injury *in vitro*, TUNEL-positive cells were visible in H/R group. Significantly less apoptosis was observed in ATRA treatment groups (*P*<0.05). We observed the same phenomenon *in vivo* (Fig 4), which showed more TUNEL-positive cells in heart tissues with I/R injury in the I/R group compared with ATRA+I/R group. TUNEL results were further confirmed using Annexin V-PI FACS staining. Our data showed significantly fewer apoptotic cells in with ARTA treatment groups than in the H/R group (Fig 3B). The key mediators of cell apoptosis, including cleaved caspase-3 and BCL-2, were also measured. As shown in Fig 5A and 5B, ATRA treatment was associated with significantly reduced cleaved caspase-3 and increased bcl-2 in H9c2 cells. This was also confirmed *in vivo* (Fig 5C). These results indicate that ATRA can protect cardiomyocytes from I/R-induced apoptosis.

### ATRA inhibited I/R-induced ROS production in H9c2 cells

**Fig 6:**
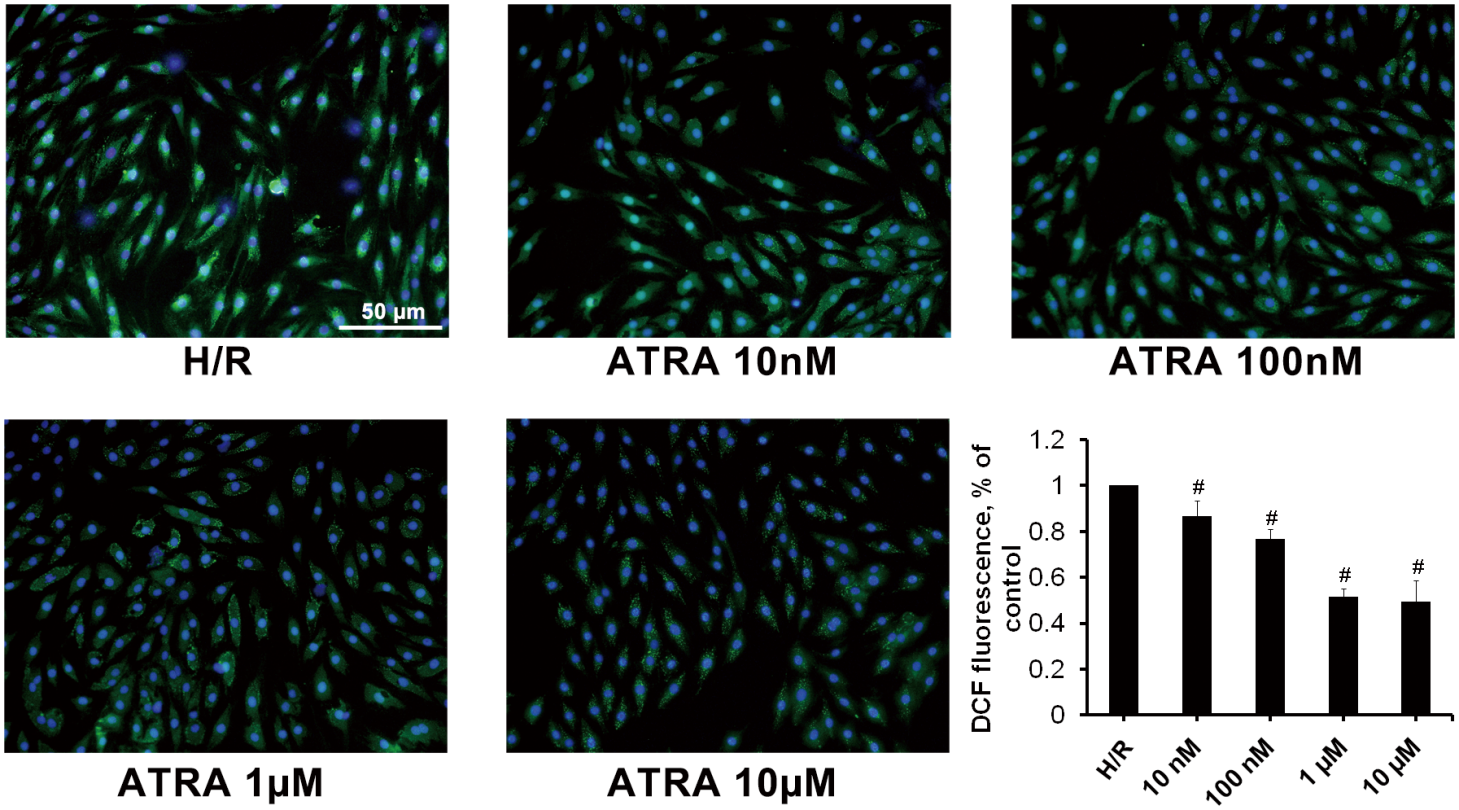
ATRA decreases ROS production in H9c2 cells after H/R injury. DCF fluorescence in H/R-induced H9c2 cells pre-treated with different concentrations of ATRA was observed using fluorescence microscopy and measured by FACS analysis (bar graph). Data is expressed as means ± SD of three independent experiments (H/R ratio was set to 1; ^#^ *P*<0.05 *vs*. H/R).

To investigate whether ATRA had anti-oxidative activity in H9c2 cells subjected to H/R, cells pre-treated with different concentrations of ATRA for 24 h and subjected to H/R injury, and ROS measured. As shown in Fig 6, treatment of H9c2 cells with ATRA led to a dose-dependent decrease in ROS levels as depicted by less DCF-positive cells.

### ATRA inhibited MAPK signaling, increased ADAM10 expression and decreased RAGE expression after I/R injury

**Fig 7:**
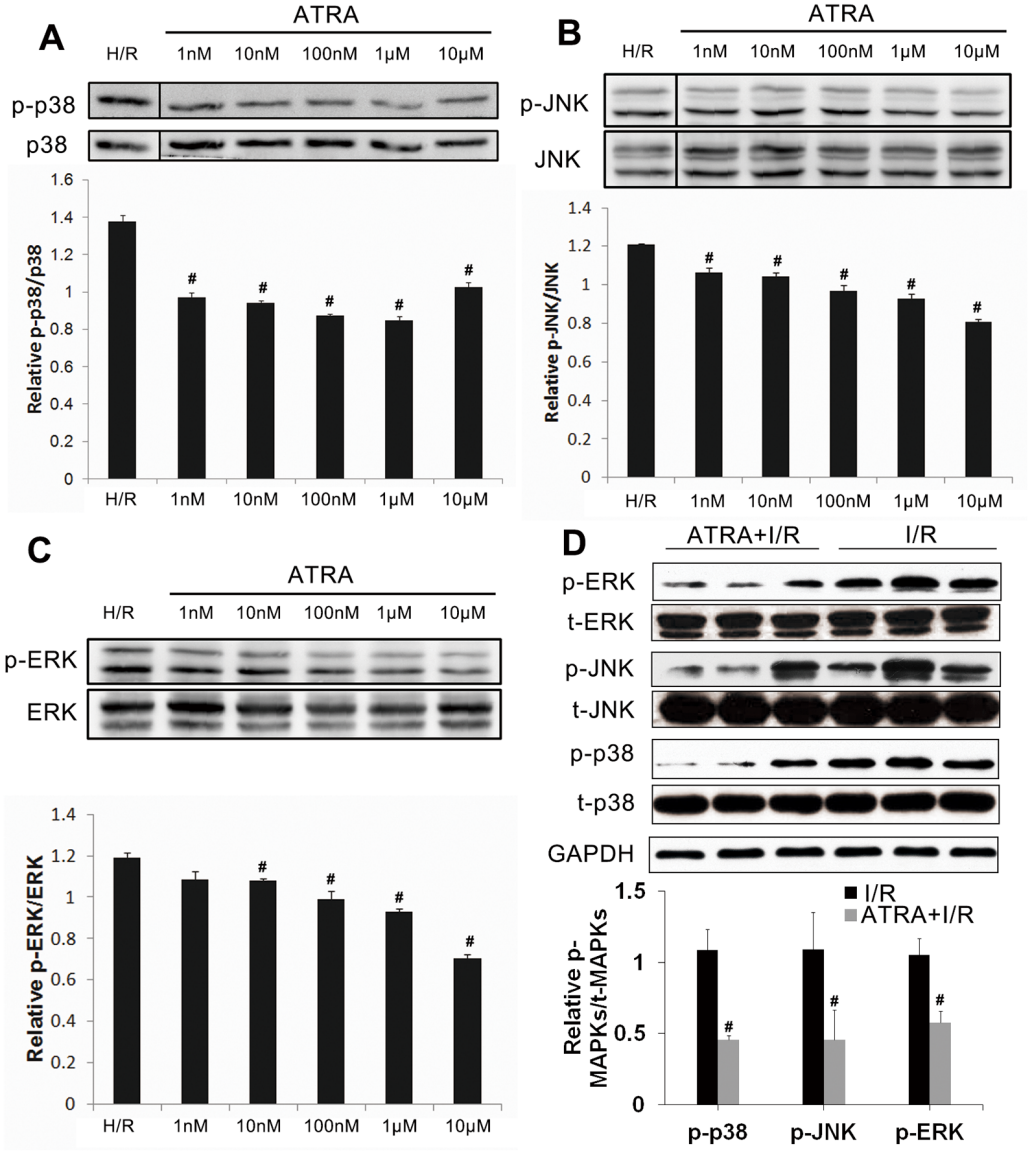
ATRA inhibits the MAPK signaling pathway in cardiomyocyte with I/R injury. (**A–C**) H9c2 cells were incubated with different concentrations of ATRA for 24 h and then subjected to H/R injury. A DMSO equivalent to ATRA at the highest concentration served as a control (H/R group). Aliquots of cell lysate were analyzed with Western blotting. ATRA inhibited phosphorylation of MAPKs, including p38 MAPK, ERK, and JNK in a concentration-dependent manner. Total MAPKs served as loading controls (n = 3 in each group, ^#^ *P*<0.05 *vs*. H/R). (**D**) The *in vitro* findings were confirmed in mouse cardiomyocytes after I/R injury. Total MAPKs served as loading controls (n = 3 in each group, ^#^ *P*<0.05 *vs*. I/R). Data is expressed as means ± SD.

Possible mechanisms underlying the protective effect of ATRA in I/R-induced apoptosis were investigated. The MAPK signaling pathway has been reported to be a main regulator of I/R-induced cell apoptosis [7]. As shown in Fig 7, both *in vitro* (Fig 7A–7C) and *in vivo* (Fig 7D) studies showed expression of p-p38, p-JNK, and p-ERK to be lower in ATRA-treated groups than in untreated groups, indicating that ATRA may protect against I/R-induced myocardial apoptosis *via* down-regulation of MAPK signaling.

**Fig 8:**
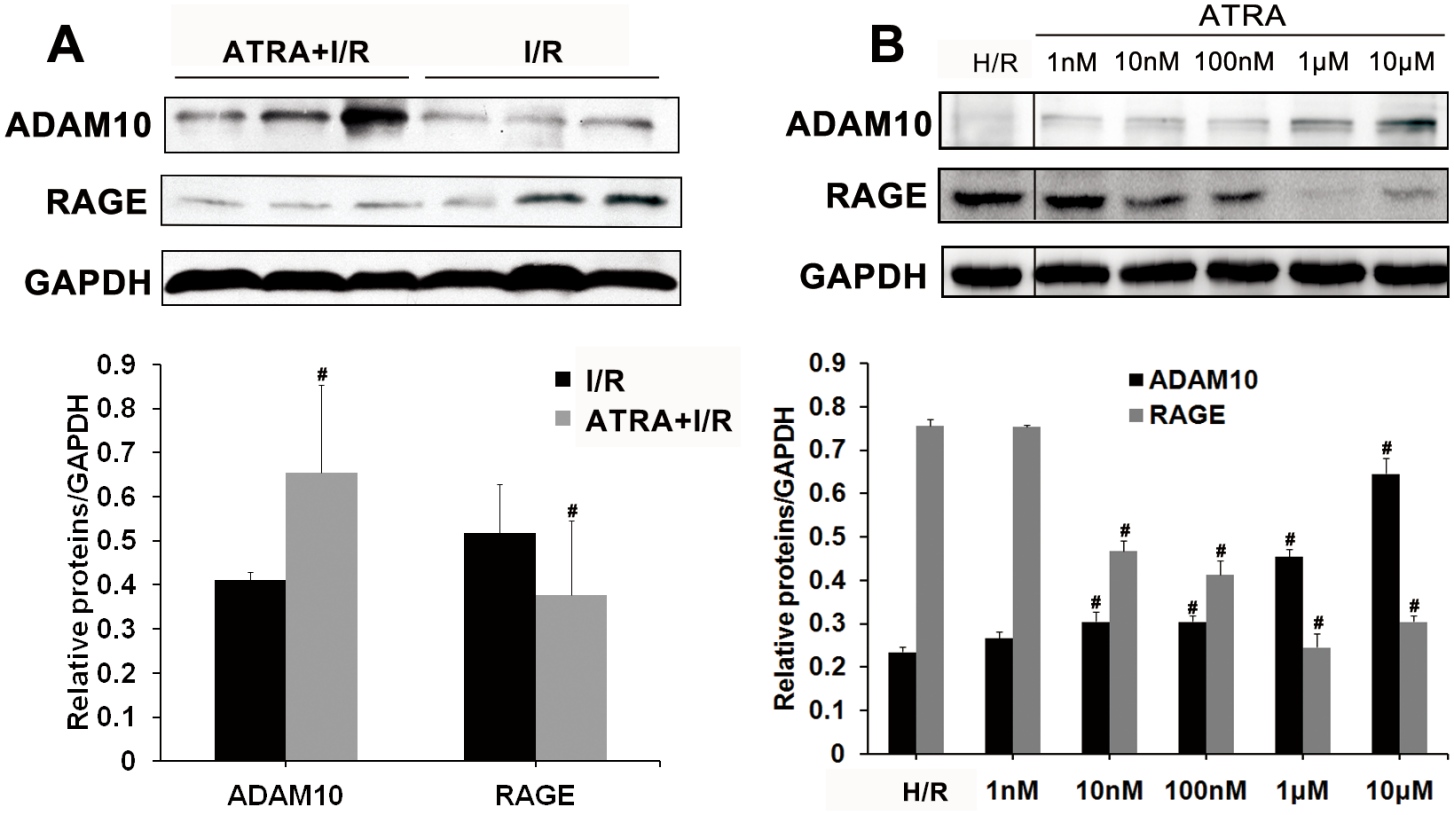
ATRA increases ADAM10 and decreases RAGE expression after I/R injury. Both *in vivo* and *in vitro* studies showed that ATRA treatment increased ADAM10 expression. Levels of RAGE, which is cleaved by ADAM10, were low. GAPDH served as a loading control (control ratio was set to 1; n = 3 in every group, ^#^ *P* < 0.05 *vs*. control). Data is expressed as means ± SD.

The levels of expression of ADAM10 and RAGE were studied to investigate the mechanisms underlying of the protective role of ATRA. As shown in Fig 8, both *in vivo* (Fig 8A) and *in vitro* (Fig 8B) studies showed that the expression of ADAM10 was higher, while the expression of RAGE was lower in ATRA-treated groups than in untreated groups. The protective role of ATRA on myocardial I/R injury was not observed in RAGE-KO mice (Fig 1).

**Fig 9:**
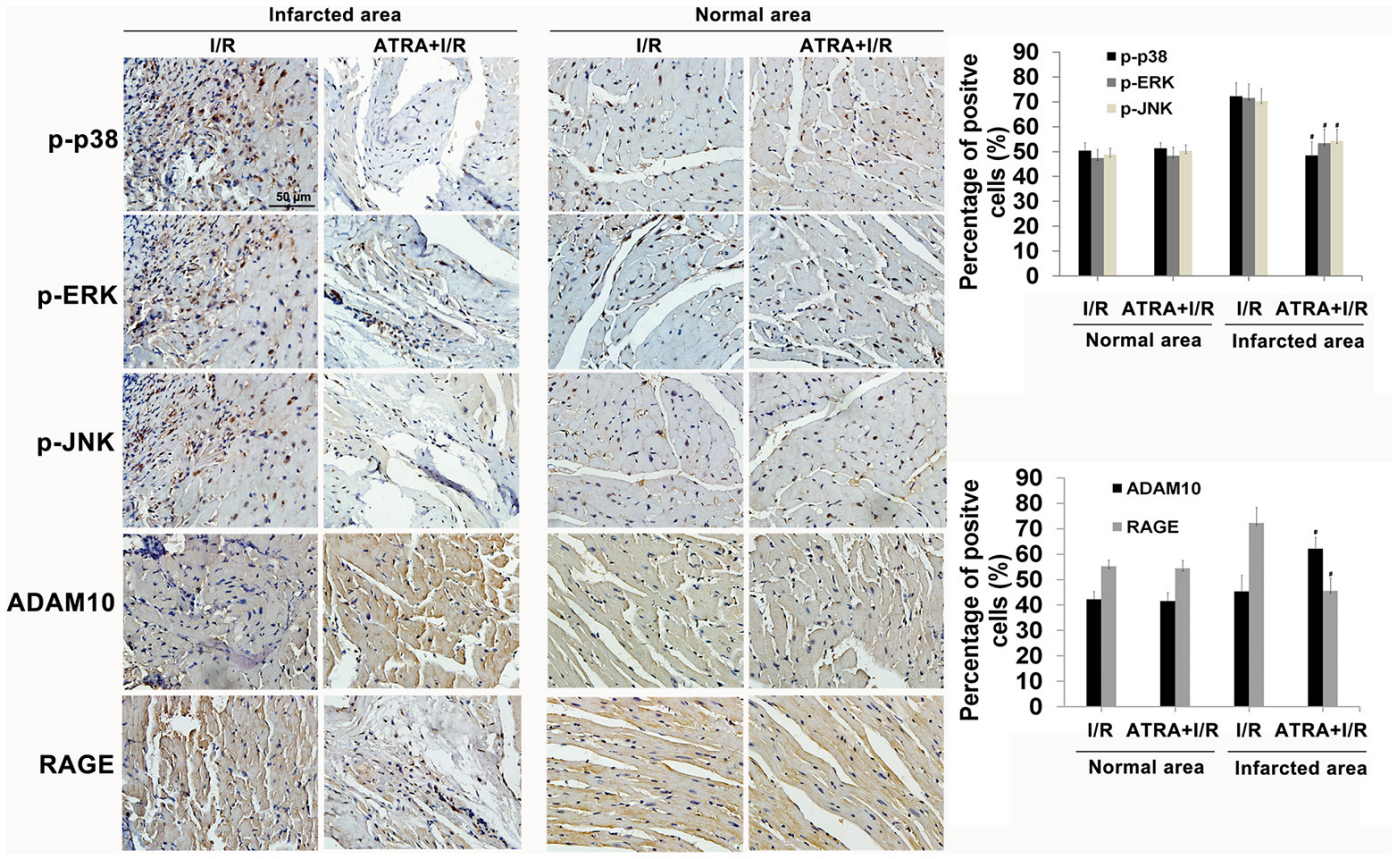
RAGE, ADAM10, and phospho-specific MAPKs expressions in non-risk (normal) and infarction area of cardiac tissues after I/R injury. Quantitative measurement of immunohistochemical images as a ratio of positive cells indicated that phospho-specific p38, phospho-specific JNK, phospho-specific ERK, and RAGE expressions were decreased, while ADAM10 expression was increased in the infarction area of the ATRA treatment group. Data is expressed as means ± SD (^#^ *P*<0.05 *vs*. I/R).

Furthermore, we assessed the expression of p-p38, p-NK, p-ERK, RAGE, and ADAM10 *via* immunohistochemistry. Data showed that ATRA treatment increased ADAM10 expression and reduced the level of RAGE and phosphorylation of p38, JNK, ERK in heart tissues with I/R injury (Fig 9), which confirm the WB analysis results.

## Discussion

Myocardial I/R injury is a complex process which ultimately leads to cell damage and organ dysfunction. Apoptosis, a form of programmed cell death, has been confirmed to occur rarely during ischemia, but it is prolific during reperfusion, according to experimental studies and clinical observations [17]. Therapies targeting apoptosis may be useful in the prevention of myocardial I/R injury. ATRA, a biologically active derivative of vitamin A (retinol), has been reported to possess biological activity including anti-apoptotic properties [18,19]. ATRA binds retinoid X receptors (RXRs) and nuclear retinoic acid receptors (RARs) to exert its protective effects, and these events have been reported in liver I/R injury [13] [20]. We report here that ATRA treatment can decrease the size of the infarct area, increase the ejection fraction after myocardial I/R injury due to its anti-apoptotic properties.

The details of the mechanisms involved in triggering apoptosis during reperfusion are unclear, but activation of MAPK signaling is thought to be responsible [21,22]. MAPK signaling involves a family of serine/threonine kinases that include three major subgroups: extracellular signal-regulated kinase [ERK], c-Jun N-terminal kinase [JNK], and p38 MAPK [p38] [23]. Previous studies have shown that activation of the MAPK signaling pathway is relevant to the regulation of myocardial apoptosis during reperfusion [7]. MAPK signaling, specifically p38 and JNK signaling, is primarily involved in induction of apoptosis during reperfusion [24–26]. Studies have shown that inhibition of apoptosis by ATRA was associated with down-regulation of MAPK phosphorylation, suggesting that ATRA may inhibit myocardial apoptosis via reduced MAPK signaling.

**Fig 10:**
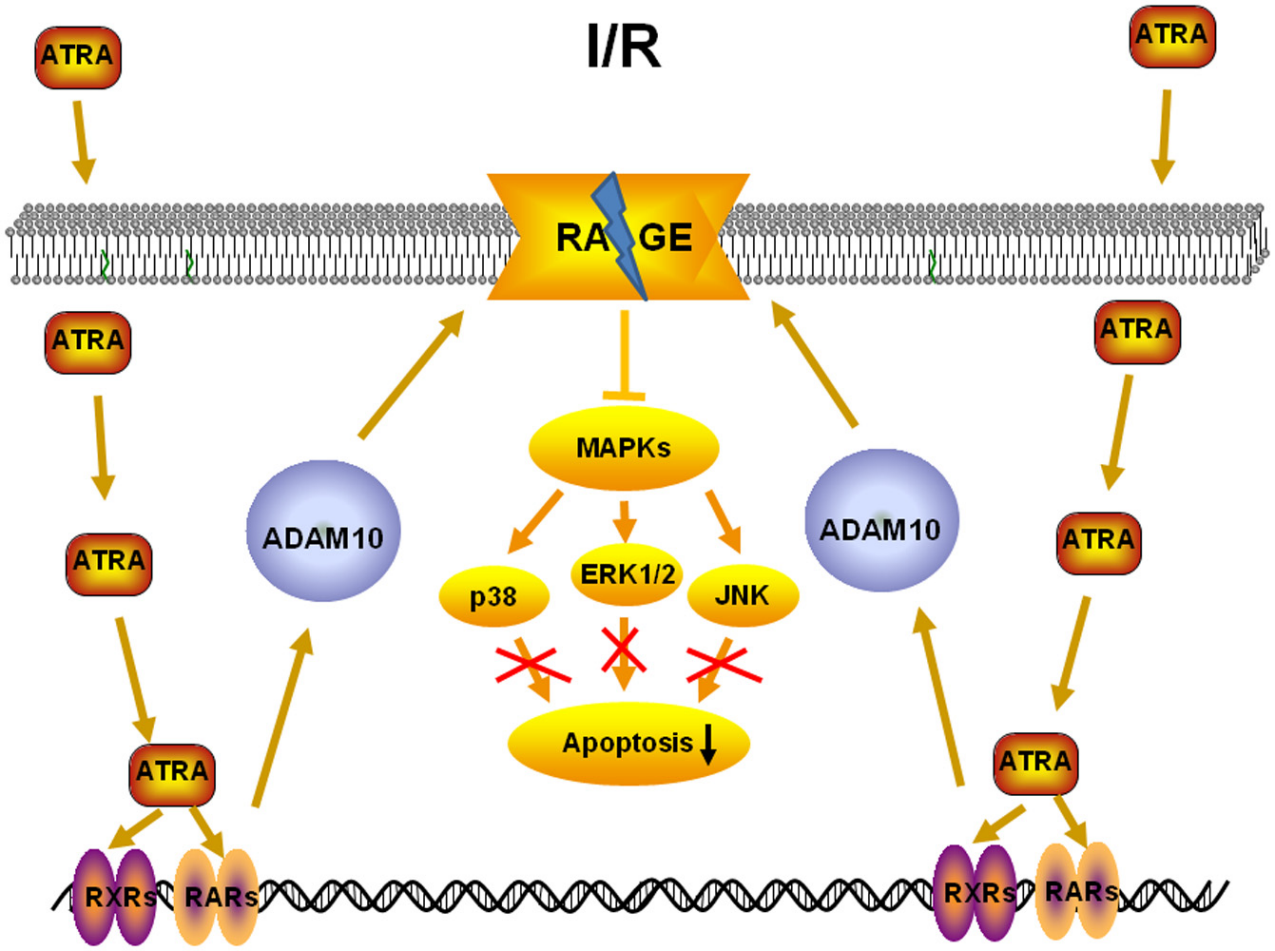
One possible mechanism underlying the effect of ATRA on myocardial I/R injury.

In response to ATRA, RARs/RXRs can bind the DNA of target genes and significantly increase their transcriptional activity. Tippmann *et al*. showed that ATRA can stimulate ADAM10 promoter activity with an EC_50_ of 1.5 μM and that this led to an increase in the amount of mature ADAM10 protein and a two- to three-fold increase of the ratio between α- and β-secretase ADAM10 activity [27]. ADAM10 primarily cleaves membrane proteins on the surface of the cell, and is also responsible for the cleavage of RAGE [28]. RAGE, which belongs to the immunoglobulin superfamily, is a multi-ligand signal transduction receptor, mediating responses to stress, including myocardial I/R injury [29]. When bound by its ligands (such as advanced glycation end products, S100/calgranulin family, high mobility group box (HMGB)-1, and integrin Mac-1), RAGE activates MAPK signaling and triggers acute and chronic inflammation, which has been observed in myocardial I/R injury [30,31]. The present study showed that ATRA treatment could increase the expression of ADAM10 and decrease that of RAGE both *in vivo* and *in vitro*. Importantly, the protective role of ATRA on cardiac I/R injury was not observed in RAGE-KO mice. These results indicate that ATRA may exert its anti-apoptotic effects *via* ADAM10-RAGE-MAPK axis (Fig 10).

Though the increase of ADAM10 expression after ATRA treatment was confirmed in the present study, the underlying mechanisms that increase ADAM10 expression to ultimately cleave RAGE and thus protect myocardiocyte from I/R injury should be further studied. Measurement of the levels of soluble form of RAGE (sRAGE), a product of RAGE cleavage, should be assessed in future studies.

## Conclusions

In conclusion, these data show that ATRA reduced cardiomyocyte apoptosis after I/R effectively and that this anti-apoptotic effect may be attributed to its ability to stimulate ADAM10, cleave RAGE, and finally down-regulate MAPK signaling.

## Supporting Information

S1 Fig ATRA protects H9c2 cells from H/R-induced cell death in a time-dependent manner. H9c2 cells were pre-treated with 1 μM ATRA for different time point and then subjected to 6 h/1 h H/R injury. The protective effect of ATRA on H/R injury was showed since the incubation time of ATRA was longer than 24 h (n = 5 in each group, ^#^ *P*<0.05 vs. H/R).
(TIF)

S2 Fig ATRA decreased NF-κB activation but had no effect on Caveolin-1 and HSP90 expressions after I/R injury. *In vivo* studies showed that ATRA treatment decreased NF-κB activation. However, Caveolin-1 and HSP90 expressions in cardiac tissues were not influenced by ATRA treatment. GAPDH served as a loading control (^#^ *P*<0.05 vs. I/R).
(TIF)

S1 Table Effects of ATRA on intracellular calcium concentration ([Ca^2+^]i) in H9c2 cells. Control refers to cells without H/R treatment, while other groups were subjected with H/R treatment. ^a^ *P*<0.05 vs. control group; ^b^ *P*<0.05 vs. H/R group.
(DOCX)
