# Supplementary figures and images for: All-Trans Retinoic Acid Ameliorates Myocardial Ischemia/Reperfusion Injury by Reducing Cardiomyocyte Apoptosis
Source: PLoS One. 2015 Jul 17;10(7):e0133414. doi: 10.1371/journal.pone.0133414 (PMC4506146; doi:10.1371/journal.pone.0133414)

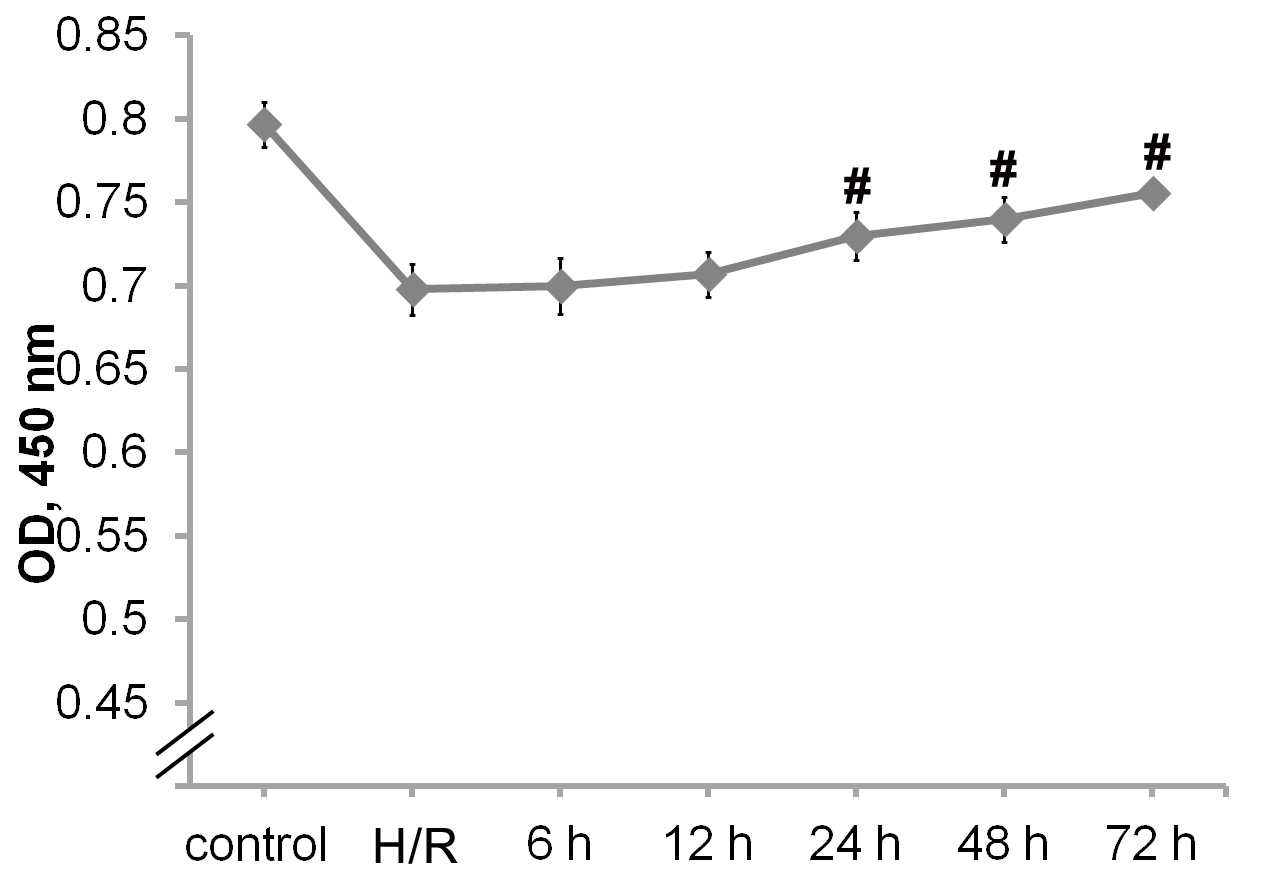

Supplement: S1 Fig — H9c2 cells were pre-treated with 1 μM ATRA for different time point and then subjected to 6 h/1 h H/R injury. The protective effect of ATRA on H/R injury was showed since the incubation time of ATRA was longer than 24 h (n = 5 in each group, # P<0.05 vs. H/R). (TIF) [file pone.0133414.s001.tif]

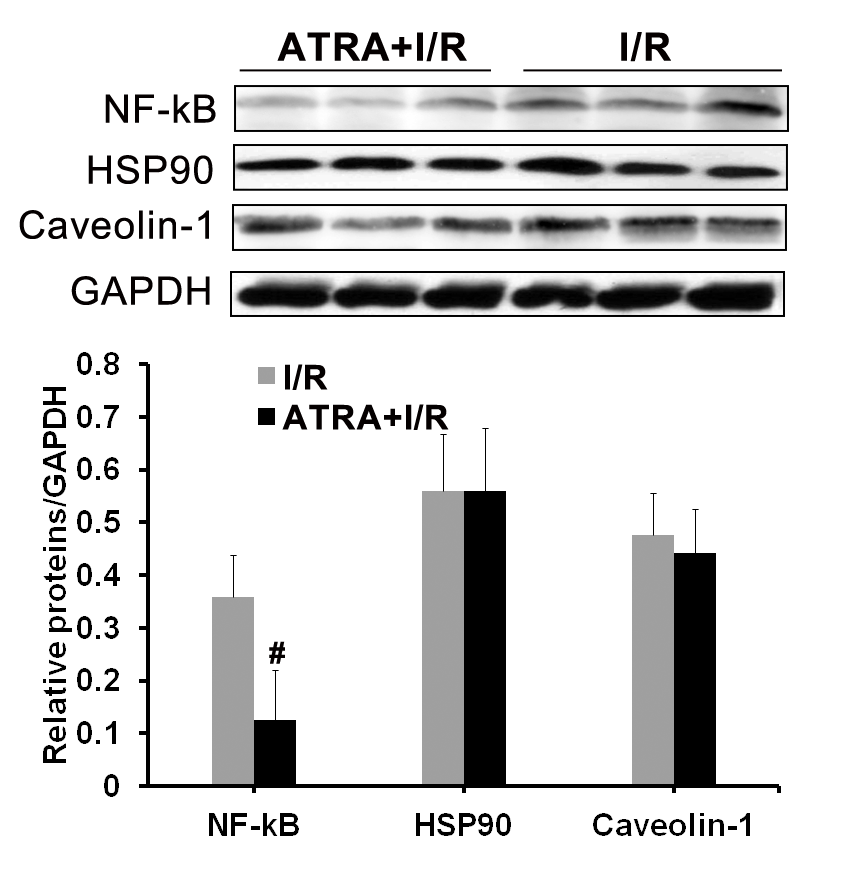

Supplement: S2 Fig — In vivo studies showed that ATRA treatment decreased NF-κB activation. However, Caveolin-1 and HSP90 expressions in cardiac tissues were not influenced by ATRA treatment. GAPDH served as a loading control (# P<0.05 vs. I/R). (TIF) [file pone.0133414.s002.tif]
