# Supplementary material for: All-Trans Retinoic Acid Ameliorates Myocardial Ischemia/Reperfusion Injury by Reducing Cardiomyocyte Apoptosis
Source: PLoS One. 2015 Jul 17;10(7):e0133414. doi: 10.1371/journal.pone.0133414 (PMC4506146; doi:10.1371/journal.pone.0133414)
Supplement: S1 Table — Control refers to cells without H/R treatment, while other groups were subjected with H/R treatment. a P<0.05 vs. control group; b P<0.05 vs. H/R group. (DOCX) [file pone.0133414.s003.docx]

**S1 Table. Effects of ATRA on intracellular calcium concentration ([Ca^2+^]i) in H9c2 cells.**

|  | Control | H/R | ATRA concentrations | | | |
| --- | --- | --- | --- | --- | --- | --- |
|  |  |  | 10 nM | 100 nM | 1 µM | 10 µM |
| (Ca^2+^)i/µM | 145.25±20.54 | 705.45±24.68^a^ | 524.19±19.47^b^ | 487.42±21.97^b^ | 319.75±12.45^b^ | 309.75±26.74^b^ |

Control refers to cells without H/R treatment, while other groups were subjected with H/R treatment. ^a^*P*<0.05 vs. control group; ^b^*P*<0.05 vs. H/R group.
